# Sharing detection heterogeneity information among species in community models of occupancy and abundance can strengthen inference

**DOI:** 10.1002/ece3.8410

**Published:** 2021-12-07

**Authors:** Thomas V. Riecke, Dan Gibson, Marc Kéry, Michael Schaub

**Affiliations:** ^1^ Swiss Ornithological Institute Sempach Switzerland; ^2^ Department of Fish, Wildlife, and Conservation Biology Colorado State University Fort Collins Colorado USA

**Keywords:** abundance, community, demography, detection, hierarchical model, multilevel model, N‐mixture model, occupancy model, Paridae, species interactions

## Abstract

1. The estimation of abundance and distribution and factors governing patterns in these parameters is central to the field of ecology. The continued development of hierarchical models that best utilize available information to inform these processes is a key goal of quantitative ecologists. However, much remains to be learned about simultaneously modeling true abundance, presence, and trajectories of ecological communities.
2. Simultaneous modeling of the population dynamics of multiple species provides an interesting mechanism to examine patterns in community processes and, as we emphasize herein, to improve species‐specific estimates by leveraging detection information among species. Here, we demonstrate a simple but effective approach to share information about observation parameters among species in hierarchical community abundance and occupancy models, where we use shared random effects among species to account for spatiotemporal heterogeneity in detection probability.
3. We demonstrate the efficacy of our modeling approach using simulated abundance data, where we recover well our simulated parameters using N‐mixture models. Our approach substantially increases precision in estimates of abundance compared with models that do not share detection information among species. We then expand this model and apply it to repeated detection/non‐detection data collected on six species of tits (Paridae) breeding at 119 1 km^2^ sampling sites across a *P*. *montanus* hybrid zone in northern Switzerland (2004–2020). We find strong impacts of forest cover and elevation on population persistence and colonization in all species. We also demonstrate evidence for interspecific competition on population persistence and colonization probabilities, where the presence of marsh tits reduces population persistence and colonization probability of sympatric willow tits, potentially decreasing gene flow among willow tit subspecies.
4. While conceptually simple, our results have important implications for the future modeling of population abundance, colonization, persistence, and trajectories in community frameworks. We suggest potential extensions of our modeling in this paper and discuss how leveraging data from multiple species can improve model performance and sharpen ecological inference.

## Graphical abstract

Multispecies models are becoming increasingly popular to understand community dynamics. In this manuscript, we demonstrate a simple technique for sharing information among species about the observation process, increasing precision and accounting for heterogeneity in detection.

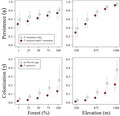

## 1 INTRODUCTION

An understanding of changes in population abundance, persistence, and colonization and the demographic processes that drive these changes is a key focus of ecology and many related fields such as conservation biology and wildlife management. Further, scaling up local demographic processes and traits to understand range‐wide population changes is a fundamental question in population and community ecology (Sutherland et al., 2013). Thus, the accurate estimation of abundance and its demographic drivers is critical. The increasing accessibility of complex ecological models (e.g., Kéry & Royle, 2021; Schaub & Kéry, 2021) and “big data,” and the introduction of novel quantitative approaches (e.g., Besbeas et al., 2002; Dail & Madsen, 2011; Royle, 2004; Zhao et al., 2017, 2019) have allowed ecologists to begin to estimate these parameters more widely and efficiently. The last two decades have seen a tremendous increase in the use and development of quantitative models to estimate the distribution, abundance, and demographic rates of populations (e.g., Hobbs & Hooten, 2015; Hooten & Hefley, 2019; Kéry & Royle, 2016, 2021; Royle et al., 2013; Royle & Dorazio, 2008; Schaub & Kéry, 2021; Williams et al., 2002), and it seems evident that the next two decades will see a tremendous increase in the application of these techniques to communities (e.g., Montaño‐Centellas et al., 2021) or groups of multiple species.

Dynamic N‐mixture and occupancy models allow for the formal modeling of demographic processes with counts or detection/non‐detection data of unmarked individuals (Dail & Madsen, 2011; MacKenzie et al., 2003; Royle & Kéry, 2007; Sollmann et al., 2015; Zipkin et al., 2014). However, a critical consideration in the effective use of N‐mixture models is unmodeled heterogeneity in detection probability (Barker et al., 2018; Duarte et al., 2018; Link et al., 2018) or ecological processes (Bellier et al., 2016). Unmodeled heterogeneity can lead to poor model fit, parameter estimates far from ecological truth, and consequently flawed inference and conservation action (Barker et al., 2018; Duarte et al., 2018; Link et al., 2018). Thus, effectively modeling patterns in detection probability and ecological processes is important for the utility of these model types (Bellier et al., 2016; Kéry & Royle, 2021).

Multispecies or community models (Dorazio & Royle, 2005; Gelfand et al., 2005, 2006; MacKenzie et al., 2005) allow for sharing information about demographic and abundance parameters among species. Recent work on N‐mixture models has examined the potential for estimation of visit‐specific heterogeneity in detection probability (Dorazio et al., 2013; Martin et al., 2011), as well as hierarchical modeling of variation in mean detection probability among species (Gomez et al., 2018). Further, previous research has suggested approaches to use information from multiple species to inform the observation process (MacKenzie et al., 2005; Nichols et al., 2000). In this study, we build on this research to estimate species‐ and visit‐specific detection probabilities in a Bayesian hierarchical framework. Our central idea is simple: *unknown and unmodeled factors that affect the detection probability of one species during surveys will often affect the detection probability of other similar species in the same way*. This is true because components that affect detection such as weather, habitat, date, time, and observer are the same during each multi‐species survey. In fact, we note that simultaneous observations of multiple species are not statistically independent observations. Thus, we use random effects to share information about heterogeneity in nuisance parameters (i.e., detection probability) among species and clearly demonstrate in a simulation study that this approach can lead to more accurate and precise estimates of model parameters when model assumptions are met.

To further demonstrate the efficacy of our approach beyond static N‐mixture models, we use our shared parameterization for the detection process with a dynamic occupancy model (MacKenzie et al., 2003; Royle & Kéry, 2007) applied to detection/non‐detection data collected on willow (*Poecile montanus montanus*, *P*. *m*. *salicarius*, and *P*. *m*. *rheananus*), marsh (*P*. *palustris*), crested (*Lophophanes cristatus*), coal (*Periparus ater*), blue (*Cyanistes caeruleus*), and great (*Parus major*) tits (Paridae) breeding across a *P*. *montanus* hybrid zone and elevational gradient in northern Switzerland (2004–2020). These six species can be loosely sorted into low and high elevation guilds in Switzerland, where marsh, blue, and great tits typically peak in abundance near 500 m in deciduous forests, and willow, coal, and crested tits typically peak in abundance near 1500 m in coniferous forests (Knaus et al., 2018). Further, tits have often been used as model organisms to examine inter‐ and intra‐specific competition (Alatalo et al., 1986; Dhondt, 1977) during the breeding (Dhondt, 2010; Minot & Perrins, 1986) and non‐breeding (Dhondt & Eyckerman, 1980) seasons. Although the majority of studies have focused on competition within the previously described elevation guilds (e.g., Alatalo et al., 1985; Wittwer et al., 2015), previous research has demonstrated that in the absence of marsh tit, willow tits occupy lower elevation deciduous habitats (Alatalo et al., 1985). As global climates warm, montane vegetation communities shift upslope (Beckage et al., 2008), albeit as a function of a variety of complex processes (Alexander et al., 2018; Scherrer et al., 2020). In response, avian communities often shift upslope as well (Freeman et al., 2018; Gasner et al., 2010; Paxton et al., 2016). Changes in the distribution and abundance of species can lead to novel competitive landscapes and communities (Alexander et al., 2015), as well as opportunities to examine the effects of interspecific competition on species distribution (Alexander et al., 2016; Legault et al., 2020). Thus, as marsh tits follow deciduous vegetation upslope, we might expect displacement of congeneric willow tits. In this study, we describe a simple multi‐species approach to improve precision of estimates of abundance and occupancy. We then demonstrate the utility of this approach by examining interspecific competition in Swiss tits, where we demonstrate that range expansion of marsh tits may lead to range contractions in willow tits, potentially affecting hybridization rates and gene flow among distinct willow tit (*P*. *montanus*) subspecies.

## 2 METHODS

### 2.1 Simulation study: estimating abundance and shared detection

We simulated data to test the efficacy of sharing observation process information among species (s) to effectively model detection parameters under a static multi‐species N‐mixture model. We first simulated population sizes (N) of five avian passerine species across 50 sites. Each species had a unique average population size corresponding to a gradient of densities, and the simulated abundance of each species (s) at each site (i) was Poisson distributed, $N_{s,i}:\text{Poisson}\left(\beta_{s}\right)$, where $\beta =\left[1,2,5,10,50\right]$. We then simulated the observation of the community (i.e., multiple species), where populations were repeatedly sampled (J=5) during each breeding season. To simulate heterogeneity in detection probability among surveys, we first simulated a shared species‐specific mean detection probability ($\bar{p}=0.5$). We simulated heterogeneity in detection ($\epsilon_{i,j}$) during each visit (j) to each site (i) that was also shared among species,

(1) $$\begin{array}{c} \epsilon_{i,j}∼N\left(0,\sigma^{2}\right),\\ \sigma =0.5. \end{array}$$

where $\epsilon_{i,j}$ are added on the logit scale and may represent unmodeled variation in observer skill and effort, local weather, date, time, and a myriad of other factors that affect simultaneously the detection of all species present. Crucially, this part of the model creates a covariation in the detection probability among the modeled species in a manner that we believe is frequently very realistic for animal surveys. The number of individuals of each species observed at each site during each visit ($y_{s,i,j}$) was simulated as a binomial trial given the number of individuals present ($N_{s,i}$) and detection probability ($p_{s,i,j}$),

(2) $$\begin{array}{c} \text{logit}\left(p_{s,i,j}\right)=\text{logit}\left(\bar{p}\right)+\epsilon_{i,j},\\ y_{s,i,j}∼\text{Binomial}\left(N_{s,i},p_{s,i,j}\right). \end{array}$$

We analyzed the multi‐species abundance data with two separate models. First, we used a classic approach to account for heterogeneity in detection (Martin et al., 2011), but treated the data for each species separately (i.e., without any shared parameters),

(3) $$\begin{array}{c} y_{s,i,j}∼\text{Binomial}\left(N_{s,i},p_{s,i,j}\right),\\ \log it\left(p_{s,i,j}\right)=\alpha_{s}+\epsilon_{s,i,j},\\ \alpha_{s}∼N\left(0,2.25\right),\\ \epsilon_{s,i,j}∼N\left(0,\sigma_{s}^{2}\right),\\ \sigma_{s}∼\text{Uniform}\left(0,1.5\right),\\ \beta_{s}∼N\left(0,100\right),\\ N_{s,i}∼\text{Poisson}\left(e^{\beta_{s}}\right) \end{array}$$

Second, we jointly analyzed the data with shared heterogeneity in detection probability among species by simplifying the estimation of heterogeneity and retaining the structure for abundance from the previous approach,

(4) $$\begin{array}{c} \text{logit}\left(p_{s,i,j}\right)=\alpha_{s}+\epsilon_{i,j},\\ \epsilon_{i,j}∼N\left(0,\sigma^{2}\right),\\ \sigma ∼\text{Uniform}\left(0,1.5\right). \end{array}$$

We simulated 100 datasets using R 4.0.3 (R Core Team, 2018), saved the medians, 95% credible intervals, and standard deviations of posterior distributions, and report mean squared error, mean signed difference and parameter calibration, or coverage (Little, 2006; Williams & Hooten, 2016) for each parameter estimated in the simulation.

### 2.2 Case study: interspecific competition in Swiss tits

To further demonstrate the use of our shared‐detection parameterization, we apply it to a community occupancy dataset to examine interspecific competition between the willow tit (*P*. *montanus*) and a congener, the marsh tit (*P*. *palustris*). We use detection/non‐detection data for willow tit, marsh tit, crested tit, coal tit, blue tit, and great tit collected at 119 $1{\;\text{km}}^{2}$ long‐term monitoring sites (i) below 1500 m in the Jura, Central Plateau, and pre‐Alps ecoregions of Switzerland. Each site was surveyed three times (j) during each breeding season (mid‐April to late June; 2004–2020; t) as part of the long‐term Swiss Breeding Bird Survey (Monitoring Häufige Brutvögel; Schmid et al., 2004).

We modeled the observation process for each species as a function of a species‐specific intercept and visit‐specific random‐effects ($\epsilon_{i,j,t}$),

(5) $$\begin{array}{c} \epsilon_{i,j,t}∼N\left(0,\sigma_{\epsilon }^{2}\right),\\ \sigma_{\epsilon }∼\text{gamma}\left(1,1\right),\\ \text{logit}\left(p_{s,i,j,t}\right)=\text{logit}\left(\alpha_{s}\right)+\epsilon_{i,j,t},\\ y_{s,i,j,t}∼\text{Bernoulli}\left(z_{s,i,t}\times p_{s,i,j,t}\right). \end{array}$$

To estimate heterogeneity in detection probability among species, sites, and surveys, we first modeled species‐specific variation in mean detection probability ($\alpha_{s}$, Gomez et al., 2018) as a function of mean detection probability of all species ($\bar{p}$) and random variation in detection probability among species ($\sigma_{\alpha }$).

(6) $$\begin{array}{c} \tau_{\alpha }=\frac{1}{\sigma_{\alpha }^{2}},\\ \sigma_{\alpha }∼\text{gamma}\left(1,1\right),\\ \bar{p}∼\text{beta}\left(1,1\right),\\ \alpha_{s}∼\text{Beta}\left(\bar{p}\tau_{\alpha },\left(1‐\bar{p}\right)\tau_{\alpha }\right). \end{array}$$

Thus, in this example, we can think of variation in species‐specific mean detection probability (α) as representing latent heterogeneity among species due to differences in visibility, behavior, or song distinctiveness and volume (Gomez et al., 2018), and in the case of occupancy models, abundance (Royle & Nichols, 2003).

We modeled the presence of each species at each 1 km^2^ survey site (Z) as Bernoulli trials. During the first occasion ($z_{s,i,1}$), we estimated presence as a function of initial occupancy probability ($\psi_{s,i}$). During subsequent occasions, we estimated occupancy ($z_{s,i,t}$) as a function of the occupancy status at the previous sampling occasion ($z_{s,i,t‐1}$), and species‐, site‐, and time‐specific persistence (ϕ) and colonization (γ) probabilities,

(7) $$z_{s,i,t}∼\left\{\begin{array}{cc} \text{Bernoulli}\left(\psi_{s,i}\right), & \text{if}\;\;t=1\\ \text{Bernoulli}\left(\phi_{s,i,t}\right), & \text{if}\;\;t>1\;\;\&\;\;z_{s,i,t‐1}=1\\ \text{Bernoulli}\left(\gamma_{s,i,t}\right), & \text{if}\;\;t>1\;\;\&\;\;z_{s,i,t‐1}=0 \end{array}\right.,$$

We modeled the initial occupancy probability of each species at each site ($\psi_{s,i}$) as a function of a species‐specific intercept ($\beta_{0,s}^{\psi }$) and linear species‐specific relationships with percent forest cover ($f_{i}$) and elevation ($e_{i}$),

(8) $$\text{logit}\left(\psi_{s,i}\right)=\beta_{0,s}^{\psi }+\beta_{1,s}^{\psi }\times f_{i}+\beta_{2,s}^{\psi }\times e_{i}.$$

We used a similar approach to model persistence (ϕ) and colonization (γ) probability for crested, coal, blue, and great tits,

(9) $$\begin{array}{c} \text{logit}\left(\phi_{s,i,t}\right)=\beta_{0,s}^{\phi }+\beta_{1,s}^{\phi }\times f_{i}+\beta_{2,s}^{\phi }\times e_{i},\\ \text{logit}\left(\gamma_{s,i,t}\right)=\beta_{0,s}^{\gamma }+\beta_{1,s}^{\gamma }\times f_{i}+\beta_{2,s}^{\gamma }\times e_{i}. \end{array}$$

Given the potential for interspecific competitive effects of marsh and willow tits, we included an effect of the presence of congeners at each site ($z_{c,i,t}$) in addition to the effects of percent forest cover and elevation,

(10) $$\begin{array}{c} \text{logit}\left(\phi_{s,i,t}\right)=\beta_{0,s}^{\phi }+\beta_{1,s}^{\phi }*f_{i}+\beta_{2,s}^{\phi }*e_{i}+\beta_{3,s}^{\phi }*z_{c,i,t‐1},\\ \text{logit}\left(\gamma_{s,i,t}\right)=\beta_{0,s}^{\gamma }+\beta_{1,s}^{\gamma }*f_{i}+\beta_{2,s}^{\gamma }*e_{i}+\beta_{3,s}^{\gamma }*z_{c,i,t‐1}. \end{array}$$

In other words, we included an intercept adjustment that accounted for the effect of marsh tit presence on willow tit persistence and colonization probability, and vice versa. We transformed covariates to standard normal deviates (i.e., forest and elevation covariates were z‐standardized). We used vague priors for slope and intercept parameters for the models of initial presence, persistence, and colonization, $\beta :N\left(0,\;100\right)$.

### 2.3 Computational implementation

We analyzed the simulated and real data using R 4.0.3 (R Core Team, 2018), JAGS (Plummer, 2003), and the jagsUI package (Kellner, 2016). We sampled using three MCMC chains for 500,000 iterations with an adaptive phase of 1000 iterations for each model for the simulated data. We discarded the first 250,000 iterations as a burn‐in and retained every fifth saved iteration. For the tit data, we sampled using three MCMC chains for 1m iterations and discarded the first 500,000 as a burn‐in. We report posterior distribution medians, 95% credible intervals, and the proportion of the posterior distribution on the same side of zero as the mean (ν) for parameters estimated in the case study. This value (ν) represents our confidence that a parameter is either greater or less than 0.

## 3 RESULTS

### 3.1 Simulation study: estimating abundance and shared detection

**FIGURE 1:**
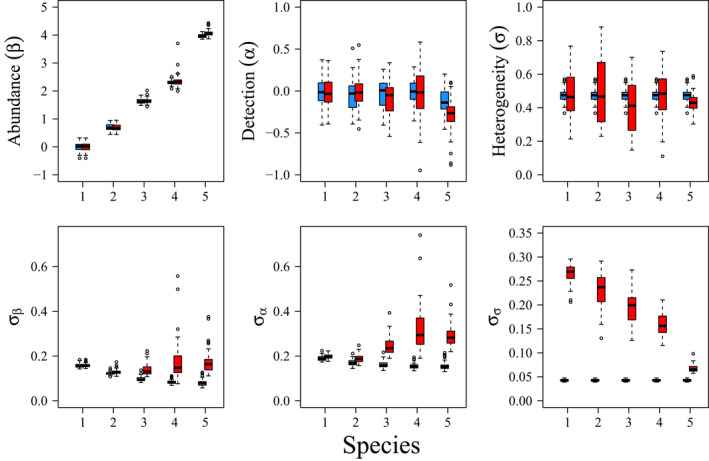
Boxplots of medians (top) and standard deviations (bottom) of posterior distributions for estimates of species‐specific mean abundance on the log‐scale (β; left), mean detection on the logit scale (α; middle), and heterogeneity in detection probability among visits (σ; right) for the simulated count data. Blue boxes and points are results from models with shared heterogeneity in detection among species, while red boxes and points are from models that analyze each species data separately. Note that joint estimates are shared among species for σ and $\sigma_{\sigma }$

Only 44 of the 100 single‐species models of simulated data converged ($\hat{R}\;<\;1.1$) with the specified MCMC settings. All of the joint analyses converged. Parameter estimates from both the joint and separate analyses were centered around the true values used to generate the data (Figure 1, Table 1). However, joint analyses substantially increased precision in estimates of abundance, mean detection probability, and detection heterogeneity among visits (Figure 1), where both mean signed difference and mean squared error were reduced compared with single‐species models (Table 1).

**TABLE 1:** Mean signed difference (MSD) and mean squared error (MSE) for the medians of posterior distributions given the true values used to simulate the data, means of the standard deviations of the posterior distributions ($\mu_{\sigma }$) from the simulations, and posterior distribution coverage for N‐mixture models with shared detection heterogeneity parameters (Joint) and models that treat each species individually (Separate)

Parameter	Joint				Separate			
	MSD	MSE	$\mu_{\sigma }$	Coverage	MSD	MSE	$\mu_{\sigma }$	Coverage
$\alpha_{1}$	−0.010	0.030	0.190	0.953	−0.008	0.032	0.198	1.000
$\alpha_{2}$	−0.033	0.034	0.171	0.953	−0.013	0.039	0.189	0.930
$\alpha_{3}$	−0.044	0.036	0.163	0.884	−0.090	0.049	0.245	1.000
$\alpha_{4}$	−0.010	0.024	0.156	0.977	−0.058	0.170	0.324	0.953
$\alpha_{5}$	−0.118	0.038	0.156	0.884	−0.293	0.127	0.298	0.884
$\beta_{1}$	−0.001	0.022	0.158	0.953	−0.003	0.023	0.159	0.953
$\beta_{2}$	0.001	0.016	0.123	0.930	−0.006	0.017	0.130	0.907
$\beta_{3}$	0.022	0.008	0.098	0.977	0.048	0.014	0.139	0.953
$\beta_{4}$	0.008	0.008	0.085	0.930	0.051	0.072	0.181	0.907
$\beta_{5}$	0.061	0.009	0.081	0.884	0.156	0.038	0.175	0.907
$\sigma_{1}$	−0.025	0.002	0.043	0.884	−0.010	0.021	0.266	1.000
$\sigma_{2}$	−0.025	0.002	0.043	0.884	−0.002	0.042	0.231	0.977
$\sigma_{3}$	−0.025	0.002	0.043	0.884	−0.091	0.037	0.195	0.977
$\sigma_{4}$	−0.025	0.002	0.043	0.884	−0.022	0.022	0.160	0.977
$\sigma_{5}$	−0.025	0.002	0.043	0.884	−0.070	0.008	0.068	0.953

Note

β is the initial abundance for each simulated species on the log‐scale, α is the intercept for detection probability of each species on the logit scale, and σ is the amount of detection heterogeneity on the logit scale.

### 3.2 Case study: interspecific competition in Swiss tits

**FIGURE 2:**
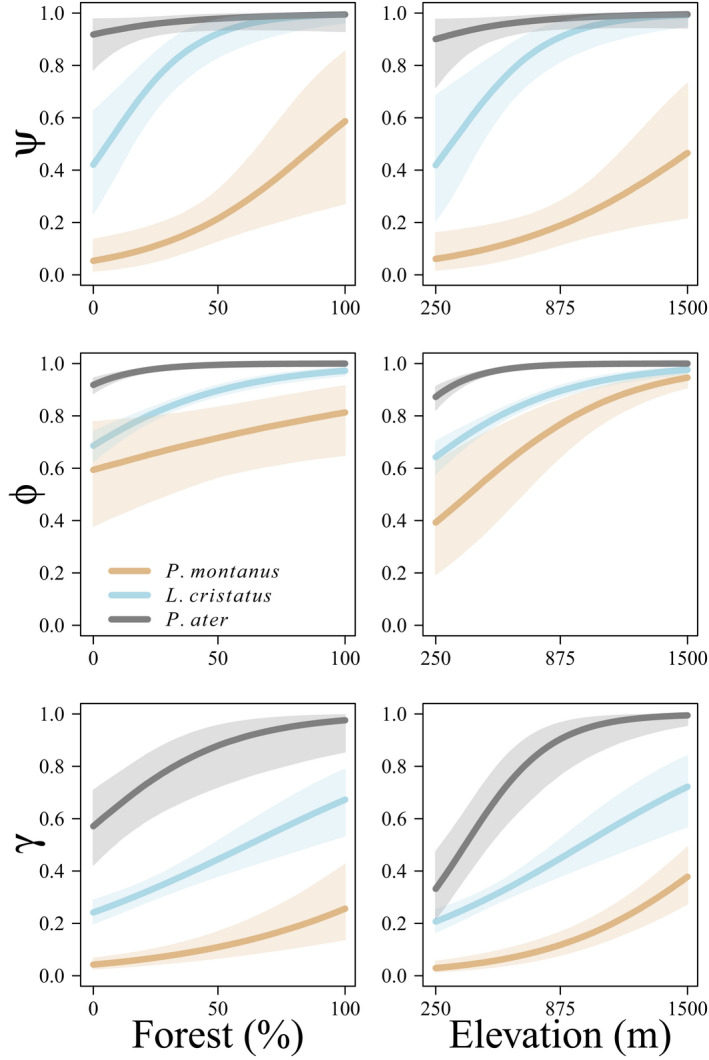
Estimated relationships between probability of presence at t=1 (ψ; top), probability of population persistence (ϕ; middle), and probability of colonization (γ; bottom) as a function of percent forest cover (left) and elevation (m; right) for willow (*Poecile montanus*; light brown), crested (*Lophophanes cristatus*; light blue), and coal (*Periparus ater*; gray) tits breeding in northern Switzerland (2004–2020)

**FIGURE 3:**
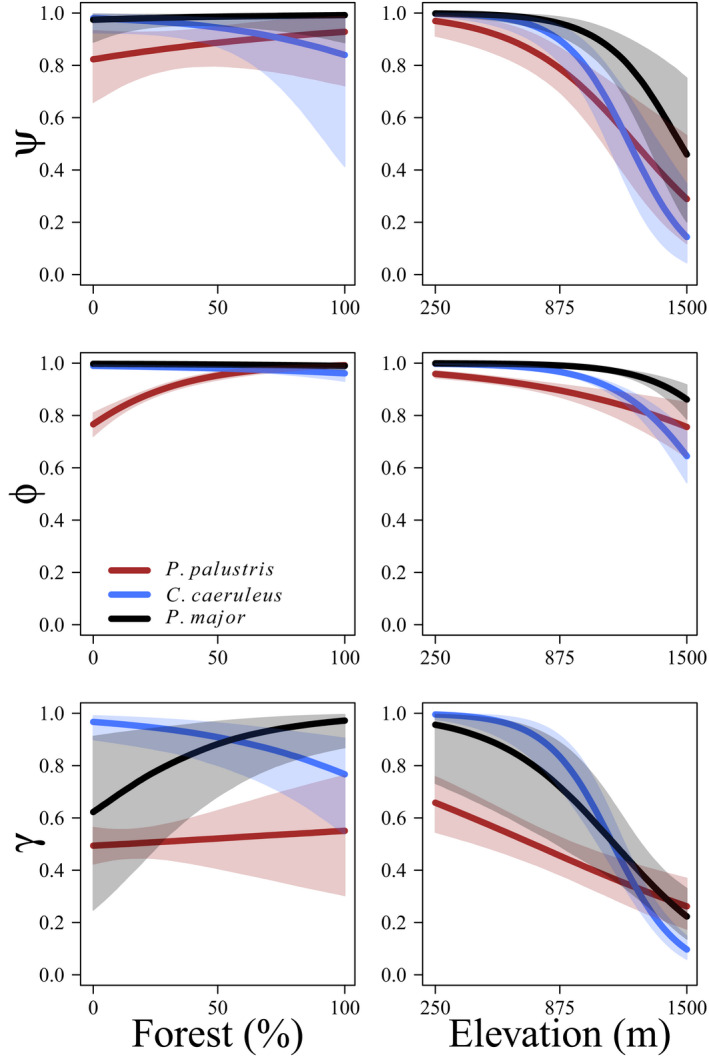
Estimated relationships between probability of presence at t=1 (ψ; top), probability of population persistence (ϕ; middle), and probability of colonization (γ; bottom) as a function of percent forest cover (left) and elevation (m; right) for marsh (*Poecile palustris*; brown), blue (*Cyanistes caeruleus*; blue), and great (*Parus major*; black) tits breeding in northern Switzerland (2004–2020)

We observed substantial heterogeneity in mean detection probability among species ($\sigma_{\alpha }=0.504;\;\;\;95\text{\textbackslash{}\%}\;\;\text{CI:}\;\;0.289‐1.060$) and shared heterogeneity in detection probability among surveys ($\sigma_{\epsilon }=0.567;\;\;\;95\text{\textbackslash{}\%}\;\;\text{CI:}\;\;\;0.460‐0.663$). As expected, we saw marked differences among species in elevation effects on tit initial occupancy (ψ), persistence (ϕ), and colonization probability (γ). Initial occupancy probability, persistence probability, and colonization probability of willow, crested, and coal tit were positively affected by elevation (Table 2, Figure 2). Initial presence probability, persistence probability, and colonization probability of marsh, blue, and great tit were negatively affected by elevation (Table 2, Figure 3). The effects of forest coverage were mixed, where only blue tits consistently demonstrated negative effects of forest cover on demographic parameters (Figure 3). Initial presence probability was positively affected by forest cover for willow, crested, and coal tits and negatively affected by forest cover for blue tits (Table 2). Forest cover positively affected persistence probability for willow, crested, coal, and marsh tits and negatively affected persistence probability for blue and great tits (Table 1). Colonization probability was positively affected by forest cover for willow, crested, coal, and great tits and negatively affected by forest cover for blue tits (Table 2).

**TABLE 2:** Medians (β), 95% credible intervals, and proportion of the posterior distribution on the same side of zero as the median (ν) for posterior distributions of the effects of percent forest cover ($\beta_{1}$) and elevation ($\beta_{2}$) on initial presence probability (ψ), persistence probability (ϕ), and colonization probability (γ) of six species of tit breeding in northern Switzerland (2004–2020)

Species	θ	Forest		Elevation	
		$\beta_{1}$ (95% CI)	ν	$\beta_{2}$ (95% CI)	ν
Willow tit	ψ	0.844 (0.119, 1.740)	0.988	0.704 (−0.001, 1.495)	0.975
	ϕ	0.285 (−0.107, 0.699)	0.923	0.894 (0.511, 1.302)	1.000
	γ	0.536 (0.262, 0.814)	0.999	0.805 (0.521, 1.093)	1.000
Marsh tit	ψ	0.267 (−0.574, 1.120)	0.739	−1.172 (−2.013, −0.493)	0.999
	ϕ	0.954 (0.642, 1.317)	1.000	−0.542 (−0.831, −0.249)	0.999
	γ	0.059 (−0.365, 0.452)	0.611	−0.452 (−0.754, −0.168)	0.999
Crested tit	ψ	1.468 (0.601, 2.808)	0.999	1.407 (0.473, 2.667)	0.999
	ϕ	0.727 (0.477, 1.001)	1.000	0.839 (0.578, 1.148)	1.000
	γ	0.487 (0.241, 0.741)	0.999	0.616 (0.310, 0.937)	0.999
Coal tit	ψ	0.715 (−0.470, 2.313)	0.868	0.863 (−0.391, 2.532)	0.899
	ϕ	1.512 (1.064, 2.066)	1.000	1.828 (1.319, 2.419)	1.000
	γ	0.898 (0.202, 1.668)	0.995	1.599 (0.753, 2.496)	0.999
Blue tit	ψ	−0.580 (−1.806, 0.524)	0.850	−2.092 (−3.257, −1.172)	1.000
	ϕ	−0.352 (−0.674, −0.023)	0.982	−1.472 (−1.852, −1.135)	1.000
	γ	−0.573 (−1.074, −0.095)	0.991	−2.049 (−2.828, −1.343)	1.000
Great tit	ψ	0.308 (−1.035, 1.804)	0.669	−1.769 (−2.952, −0.737)	0.999
	ϕ	−0.380 (−0.832, 0.077)	0.946	−1.532 (−2.127, −1.036)	1.000
	γ	0.776 (0.017, 1.615)	0.977	−1.180 (−2.196, −0.306)	0.995

**FIGURE 4:**
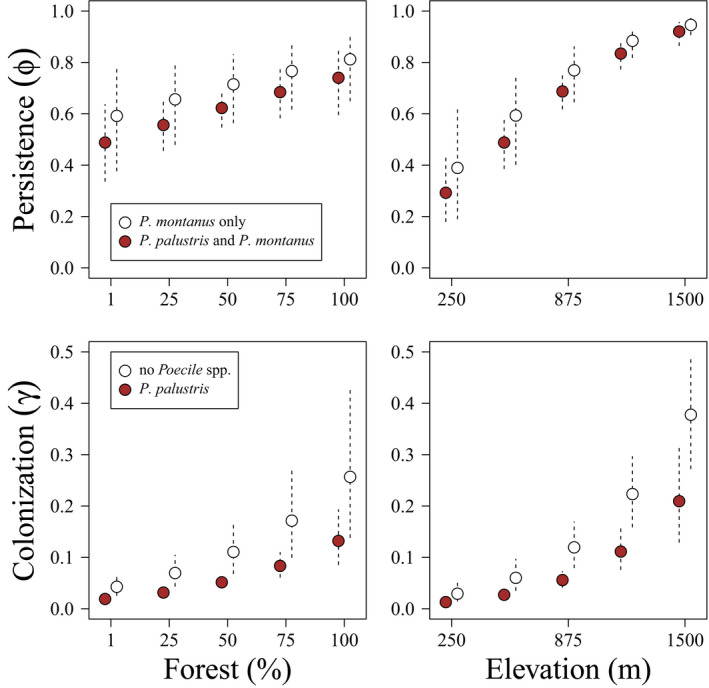
Persistence (ϕ; top) and colonization (γ; bottom) probability for willow tits (*P*. *montanus*) in the presence (brown) or absence (white) of marsh tit (*P*. *palustris*) as a function of percent forest cover (left) and elevation (m; right) in northern Switzerland (2004–2020). The points are the posterior distribution medians, and the vertical dashed lines show the 85% Bayesian credible intervals

In sites occupied by both marsh and willow tits, we found weak evidence for a decline in persistence probability of willow tits ($\beta_{3}^{\phi }=‐0.418,\;\;\;\upsilon =0.820$; Figure 4), and no effect on persistence probability of marsh tits ($\beta_{3}^{\phi }=0.242,\;\;\;\upsilon =0.734$). In contrast, we found strong evidence that marsh tit occupancy was associated with reduced colonization by willow tits ($\beta_{3}^{\gamma }=‐0.831,\;\;\;\upsilon =0.980$; Figure 4) and willow tit occupancy with reduced colonization probability by marsh tits ($\beta_{3}^{\gamma }=‐1.292,\;\;\;\upsilon =0.999$).

## 4 DISCUSSION

In many statistical community models, researchers focus on understanding interspecific effects on occupancy, abundance, and demography. In this paper, we demonstrate the importance and utility of sharing information among species to inform the observation process, thereby strengthening our inferences about the demographic processes. First, we use this approach with simulated count data where we perfectly meet model assumptions. Our results demonstrate that sharing information about heterogeneity in detection probability among species can be an effective tool for improving the precision and accuracy of detection estimates. We then apply this approach to more complex, dynamic occupancy data on tits breeding at lower elevations (<1500m) in northern Switzerland. We use data from all six tit species breeding in northern Switzerland to inform detection probability and habitat relationships, while constraining inference regarding interspecific competition to the two most closely related species, marsh and willow tit.

In northern Switzerland, marsh tits occupy lower elevation sites, while three subspecies of willow tit occupy higher elevation habitats (Knaus et al., 2018). Previous research in island systems has demonstrated that marsh tits may competitively exclude willow tits from lower elevation deciduous forests (Alatalo, Gustafsson, Lundberg, et al., 1985). In response to climate warming, montane vegetation (Beckage et al., 2008) and avian communities (Freeman et al., 2018; Gasner et al., 2010; Paxton et al., 2016) move upslope. Our results demonstrate that the presence of either species reduces the probability of colonization by the other (Table 2), and we found weak evidence ($\beta_{3}^{\phi }=‐0.430,\;\;\upsilon =0.825$) that the presence of marsh tits reduces the persistence probability of willow tit populations (Figure 4). However, over time, even a weak tendency toward displacement of willow tit by marsh tit may lead to range reduction and reduced interbreeding among willow tit subspecies (Knaus et al., 2018).

The primary tenet of the approach we describe is that detection probabilities of all surveyed species positively covary to some degree, and ideally strongly so. We expect that such positive covariation is common when species are sampled using the same protocol and do not have diametric behavior. For instance, the tit populations in this study are most often detected acoustically and singing activity commonly depends on weather, date, time of day, or habitat. If species are included that differ radically in behavior (e.g., tits and owls), we would not expect positive covariation of detection parameters. Readers should note that major violations of model assumptions will lead to incorrect parameter estimates in all hierarchical models, and these issues can be particularly problematic when using the various classes of N‐mixture models (Barker et al., 2018; Duarte et al., 2018; Link et al., 2018). Thus, we suggest that more complex and flexible random‐effects structures may be employed in the future as well. For instance, researchers might employ covariance approaches, estimating correlations among detection probabilities as a function of shared traits (see Nichols et al., 2000) or even phylogeny (e.g., Abadi et al., 2014). Researchers might also use informative priors to inform covariance in processes among species, or explicitly estimate correlations (Riecke et al., 2019). The approach described herein also allows for the simple inclusion of survey‐specific covariates, such as survey date, local weather covariates, or nesting visit‐specific random effects within observer‐specific random effects. While fully shared observation parameters may seem to be too generalized, we emphasize that this has been a hidden assumption of every existing community modeling framework that does not explicitly account for the observation process. Finally, given the strong linkage between abundance and detection of species presence (Royle & Nichols, 2003), we urge caution when using these model types with detection/non‐detection data for groups of species with extreme variation in abundance.

The estimation of abundance, distribution, and patterns in ecological communities is of central importance to a wide number of ecological fields, and continued advances in the estimation of these ecological processes will be critical to the progression of ecological knowledge (Hooten & Hefley, 2019; Hooten et al., 2017; Kéry & Royle, 2016). While static and dynamic N‐mixture models can be sensitive to model assumption violations (Barker et al., 2018; Link et al., 2018), these models can estimate abundance and demographic parameters when detection heterogeneity is adequately modeled (Ficetola et al., 2018; Kéry, 2018; Royle, 2004; Veech et al., 2016). Numerous studies have recently demonstrated methods to integrate other data types with N‐mixture models to improve performance. For example, telemetry (Ketz et al., 2018; Popescu et al., 2017; Schmidt et al., 2015), band‐recovery (Zhao et al., 2019), and genetic (Furnas et al., 2018) data have all been used to help inform demographic parameters of single species in integrated modeling frameworks. In this paper, we use the statistical dependence of community‐level count and detection/non‐detection data to inform heterogeneity in detection probability. Further, we suggest that linking the observation process among species may even allow for the inclusion of other data types (e.g., capture–recapture) from a single species to inform the observation of communities. While we urge continued caution and further simulation‐based validation of new techniques, we suggest that the thoughtful sharing of information among species within community‐level models (Gomez et al., 2018; MacKenzie et al., 2005; Nichols et al., 2000, this paper) has strong potential to improve ecological inference moving forward.

## CONFLICT OF INTEREST

There are no conflict of interest to declare.

## AUTHOR CONTRIBUTION

**Thomas V. Riecke:** Conceptualization (equal); Formal analysis (lead); Writing – original draft (lead); Writing – review & editing (equal). **Dan Gibson:** Conceptualization (equal); Formal analysis (supporting); Writing – review & editing (equal). **Marc Kéry:** Formal analysis (supporting); Supervision (supporting); Writing – original draft (supporting); Writing – review & editing (equal). **Michael Schaub:** Formal analysis (supporting); Supervision (lead); Writing – original draft (supporting); Writing – review & editing (equal).

## Data Availability

R script for simulating and analyzing the data and the data used in this manuscript are archived at the Dryad Digital Repository (https://doi.org/10.5061/dryad.573n5tb8s).
